# Atlantoaxial Subluxation Related to Axial Spondylarthritis: A Case-Based Systematic Review

**DOI:** 10.31138/mjr.070624.asr

**Published:** 2024-12-31

**Authors:** Maroua Slouma, Soumaya Rezgui, Houssem Tbini, Achraf Abdennadher, Mohamed Dehmani Yedeas, Lamjed Msolli, Khalil Amri, Leila Metoui, Rim Dhahri, Imen Gharsallah

**Affiliations:** 1 Department of Rheumatology;; 2 Department of Orthopaedic Surgery;; 3 Department of Neurosurgery;; 4 Department of Radiology, Military Hospital, Tunis, Tunisia,; 5 University of Tunis El Manar, Tunisia

**Keywords:** axial spondylarthritis, atlantoaxial subluxation, joint dislocation, joint instability, review, spondyloarthritis

## Abstract

**Aim:**

Atlantoaxial dislocation is a loss of stability between the atlas and axis. It is rarely reported in patients with axial spondylarthritis. We present an axial spondylarthritis case revealed by atlantoaxial subluxation. Case Report: We report the case of a 30-year-old man diagnosed with ankylosing spondylitis (AS) after being admitted to our department for acute atlantoaxial subluxation-related symptoms.

**Methods:**

We conducted a literature review according to the Preferred Reporting Items for Systematic Reviews and Meta-Analyses (PRISMA) guidelines using the MEDLINE database, including case reports and case series of atlantoaxial dislocation in axial spondylarthritis patients.

**Results:**

We included 16 articles. There were 134 patients (including our case), mainly male (n=119). The mean age was 34.43±12.96 years. Atlantoaxial dislocation revealed axial spondylarthritis in 4 cases. The main clinical manifestations were neck pain (12 cases), limb weakness with numbness (7 cases), cervical range of motion limitation (6 cases), neck stiffness (4 cases), muscle dystonia (2 cases), and dyspnoea (1 case). Specific neurologic signs were found in 4 patients. The atlantoaxial dislocation was anterior in 118 cases, rotatory in 5 cases, lateral in 1 case, and posterior in 1 case. Surgical treatment was the preferred option in most cases, consisting of C1-C2 arthrodesis. Outcomes were not detailed in 121 cases and were favourable for the rest. Only one patient died following a recurrence of spinal cord compression.

**Conclusion:**

Physicians need to be aware of atlantoaxial dislocation, as it could lead to spinal cord compression, vascular compression, and other serious life-threatening complications that may require surgical management.

## INTRODUCTION

Axial spondylarthritis (AxSpA), encompassing ankylosing spondylitis (AS) also referred to as radiographic AxSpA (rAxSpA) and non-radiographic AxSpA (nrAxSpA), is a chronic inflammatory disease typically affecting the spine and sacroiliac joints.

It can also be responsible for peripheral manifestations, hip involvement,^1^ and extra-articular manifestations such as uveitis, psoriasis, and bowel disease.

Clinical and radiological features in AxSpA are mainly explained by synovitis and enthesitis.^2,3^

AxSpA is characterised by inflammation, erosion, and new bone formation, affecting sacroiliac joints and the spine.^4^ It is responsible for back pain, spinal stiffness, and spine deformity.^4^

All segments of the spine can be affected in patients with AxSpA. However, the atlantoaxial joint is rarely affected. This joint can be involved in other rheumatic diseases.^5^

Atlantoaxial instability and atlantoaxial dislocation (AAD)^6^ can be due to several causes, such as trauma,^7^ infection,^8^ inflammatory diseases,^9^ or congenital anomalies.^10^ It can also be idiopathic.^11^

AAD has been described in approximately 25% of patients with rheumatoid arthritis.^12,13^ Nevertheless, it has rarely been reported in patients with AxSpA.^14^

AAD is scarce, but it can be potentially fatal. Not treated timely, it can cause permanent neurological deficits and sagittal deformity.

The aim of this systematic review was to summarise the clinical and radiological findings and therapeutic management of AAD related to AxSpA. We also report the case of a patient with AxSpA revealed by an atlantoaxial subluxation (AAS)

## METHODS

### Case report

We report a case of AxSpA revealed by an AAS. The following data were extracted from the patient's chart: demographic characteristics, clinical presentation, investigations, and therapeutic management.

The patient gave written informed consent for the publication of this case report.

### Systematic review

We followed the Preferred Reporting Items for Systematic Reviews and Meta-Analyses (PRISMA) guidelines to identify, screen, select, and include papers. The case report was presented in accordance with the CARE standard of the EQUATOR Network.^15^

#### Publication search

We performed a literature search up to November 2022 in MEDLINE for English-language sources using the following keywords chosen from the Medical Subject Headings (MeSH) of MEDLINE: (Spondylitis, Ankylosing [MeSH Terms]) AND (Atlanto-Axial Joint [MeSH Terms]); (Spondylitis, Ankylosing [MeSH Terms]) AND (Joint Dislocations [MeSH Terms]); (Spondylitis, Ankylosing [MeSH Terms]) AND (Joint Instability [MeSH Terms]). The studies were chosen for the review based on inclusion and exclusion criteria. We also conducted a manual search of the reference lists of articles identified in the initial survey to minimise the risk of missing relevant articles. The screening of titles and abstracts for eligibility were done independently by two authors. Any discrepancies were resolved through a consensus discussion with a third author.

### Inclusion criteria

We included case reports and case series describing AAD in AxSpA patients. Only English publications were included.

### Non-inclusion and exclusion criteria

We did not include cases of AAD due to other causes, such as traumatism, infection, and rheumatoid arthritis. We excluded non-full-text articles, child patients, and cases of AAD in AxSpA patients related to other causes like trauma.

### Data collection

Full-text articles from eligible abstracts were retrieved and assessed to determine whether they answered the research questions and met the inclusion criteria. The following data were collected from each case report: gender, age at diagnosis, the delay between the diagnosis of AAD and AxSpA, symptoms, imaging features, treatment, and outcomes.

## RESULTS

### Case presentation

A 30-year-old man complained of acute severe neck pain associated with paraesthesia in his arms and legs. He also reported a 9-month history of low back and neck pain, for which he self-medicates with nonsteroidal anti-inflammatory drugs (NSAIDs). Nevertheless, he denied any history of trauma and had no medical history of psoriasis or uveitis. He works as a waiter in a coffee shop.

**Figure 1.**
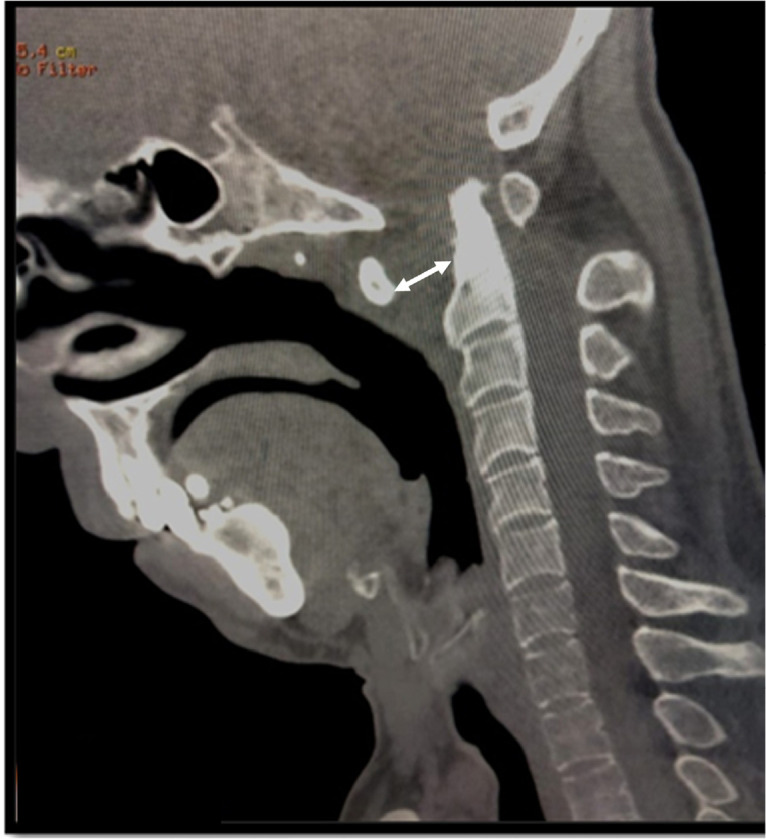
Sagittal section of cervical spine CT scan showing a C1-C2 diastasis of 15mm, suggesting an anteroposterior subluxation with a rupture of the transverse ligament.

The patient had a body mass index of 26 kg/m^2^. His body temperature was 37° Celsius. Physical examination showed restricted neck movement in the sagittal and axial planes. Deep tendon reflexes and muscle testing were normal. There were no sphincter disturbances, lower cranial nerve dysfunction, or respiratory distress. The physical examination was otherwise unremarkable. Laboratory examinations showed a slight increase in inflammatory biomarkers: the C-reactive protein (CRP) was 16 mg/L (Normal value [NV] <8 mg/L), the erythrocyte sedimentation rate (ESR) was 57 mm, and the alpha-2-globulin was 12 g/L (NV: 7.3–11 g/L). Liver tests and renal function were within normal ranges. Magnetic resonance imaging (MRI) of the cervical spine was indicated. However, the patient did not tolerate the supine position. A cervical computed tomography (CT) scan showed a 15 mm C1-C2 diastasis, suggesting an anteroposterior dislocation with transverse ligament rupture with spinal cord compression at the C1-C2 level (**Figure 1**).

**Figure 2.**
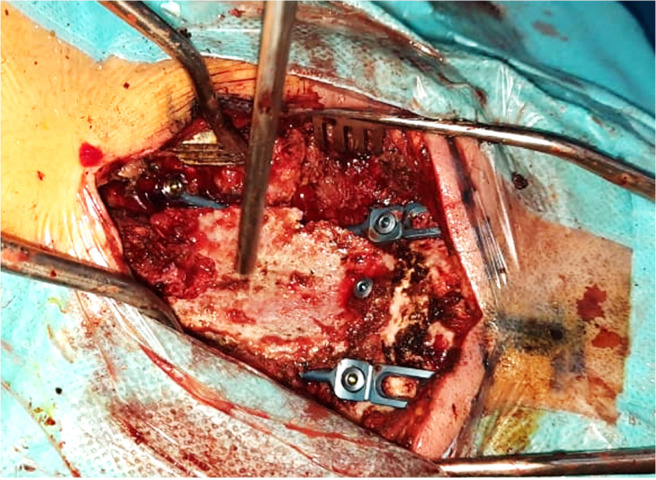
Per operative view of the posterior iliac crest crafting.

**Figure 3.**
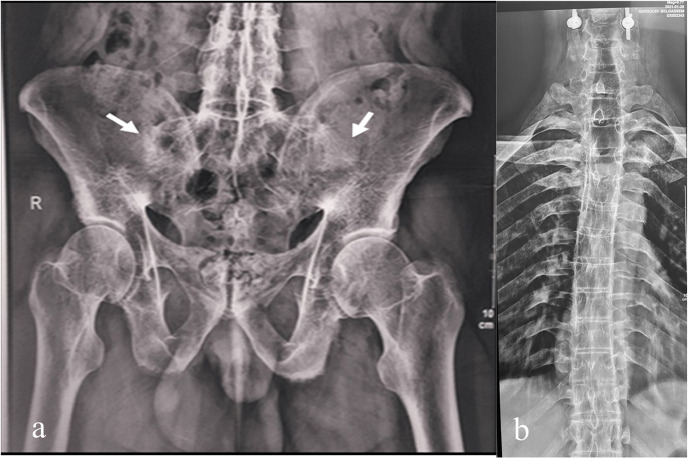
Anteroposterior radiograph of the pelvis (a) showing the total ankylosis of sacroiliac joints (arrows). Anteroposterior radiograph of the thoracolumbar spine (b) showing a complete fusion of the bones of the spine resulting in the appearance of the bamboo spine sign.

The patient underwent urgent surgery consisting of posterior decompression by suboccipital craniectomy and C1-C2 laminectomy. Iliac crest bone was previously prepared and then grafted into the C3 spinous process and occipital bone (**Figure 2**). A cervical collar was then indicated for three months. Pelvic and spine radiographs revealed bilateral ankylosis of the sacroiliac joints (**Figure 3A**) and a bamboo spine (**Figure 3B**). The diagnosis of AAD revealing ankylosing spondylitis (AS) was made based on the Assessment of SpondyloArthritis International Society (ASAS) classification criteria.^16^ The patient had high disease activity with an Axial spondylarthritis Disease Activity Score^17^ (ASDAS) at 4. He was started on infliximab at 5mg/kg at weeks 0, 2, and 6, and then every 8 weeks. After 12 months of follow-up, inflammatory biomarkers became within normal range, and ASDAS fell to 3.

### Systematic literature review

**Figure 4.**
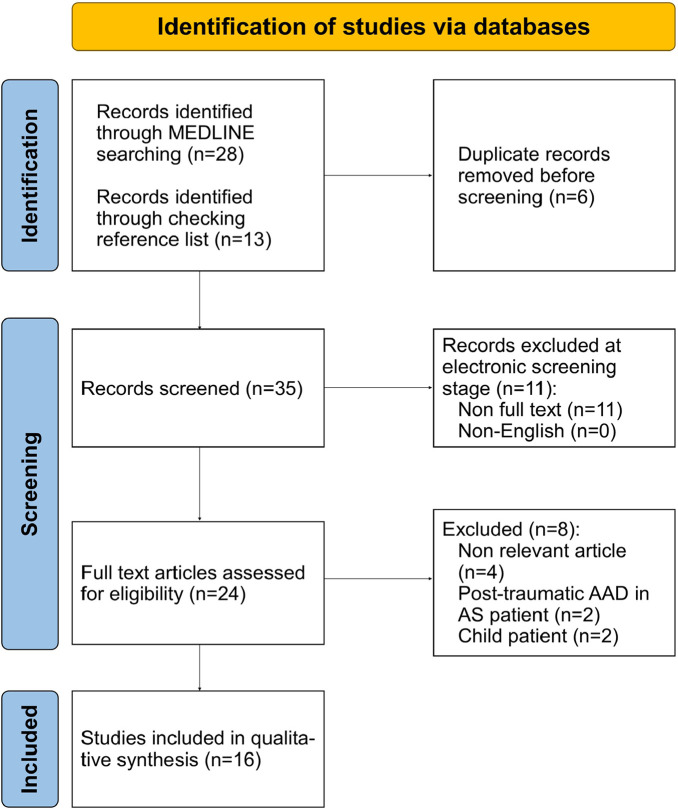
Flow chart for the study selection process.

The initial search yielded 28 articles. Non-relevant articles were removed, as shown in the flow chart (**Figure 4**). We also excluded 2 cases of post-traumatic AAD in AxSpA patients.^18,19^ The identified literature cases, including our case, are shown in **Table 1**.^20–35^ There were 134 patients: 119 men, 14 females, and not specified in one case.^21^

**Table 1.** Demographic and clinical characteristics of the included studies

**Authors**	**No. of cases**	**Gender (age in years)**	**Delay between AS diagnosis and AAD (years)**	**Clinical features**	**Type of AAD/Radiological features**	**Treatment**	**Outcome**
Stammers et al.^20^	1	Male (30)	1	Curvature of the spine Neck stiffness Weakness and numbness of the right leg Tingling and weakness of the left forearm and hand	AAS No evidence of fracture of the odontoid process	Initial fixation of the head and neck with a plaster cast in a flexed position for 3 months, followed by a leather collar	Disappearance of symptoms and neurological signs.
Wilkinson et al.^21^	1	NS	NS	Neck pain	AAS	NS	NS
Leventhal et al.^22^	1	Male (35)	3	Neck stiffness and pain Limitation of rotation and lateral tilt of the head	Rotatory atlantoaxial subluxation	Initial skeletal traction to reverse the deformity then occipito-cervical fixation	Immediate resolution of neck pain. Solid arthrodesis of the occiput at C2. Correction of rotatory and lateral tilt deformity.
Hamilton et al.^23^	1	Female (27)	0.3	Neck pain Tingling in fingers Arm heaviness	AAS; AADI=9 mm	Posterior C1-C2 fixation	Disappearance of symptoms. Radiographic confirmation of fusion three months postoperatively.
Das et al.^24^	1	Female (61)	2	Neck pain Nonreducible rotational head tilt Progressive quadriparesis	Lateral subluxation Cord compression	Occipito-cervical fixation	Significant improvement in quadriparesis and neck pain postoperatively. Residual upper-extremity numbness.
Chien et al.^25^	1	Male (49)	30	Severe torticollis Hypoglossal nerve palsy Hyperreflexia Decreased muscle strength Babinski sign	Extensive erosion of the C1-C2 facet joints with marked rotatory subluxation Substantial cervicomedullary compression	Halo-dependent traction followed by occipito-cervical fixation	Rapid resolution of symptoms. Solid occipito-cervical fusion after 2.5 years of follow-up.
Weigel et al.^26^	1	Male (46)	20	Neck pain Fixed rotation of the head at 45° to the left Dystonia in the right sternocleidomastoid and the left posterior neck muscles	Rotatory atlantoaxial subluxation and craniocervical osseous fusion	Partial myectomy of the right sternocleidomastoid muscle and selective posterior ramisectomy of C1–C6 including C2 ganglionectomy on the left side	Considerable improvement in neck pain. The rotational fixation of the head remained unchanged. The benefit was maintained two years after the surgery.
Chen et al.^27^	1	Male (23)	3	Limited range of motion of the cervical spine Intermittent arm numbness	AAS; AADI=20 mm Spinal cord compression Bone marrow oedema in the dens of the axis Periodontoid pannus	C1-C2 fixation	NS
Lui et al.^28^	3	Female (18) Male (29) Female (19)	Concomitant	Inflammatory neck pain Neck stiffness	AAS; AADI=6 mm AAS; AADI=3 mm AAS; AADI=6 mm	C1-C2 fixation	Marked improvement in neck pain after surgery. NS for 2 cases.
Rajak et al.^29^	1	Male (32)	14	Severe, intractable neck pain radiating to the occiputs and down the cervical spine	AAS; AADI=6 mm Intraspinal pannus formation and synovitis around the odontoid peg without neurological compression.	Occipito-cervical fixation	Uncomplicated postoperative recovery. Treatment with tumour necrosis factor inhibitor was delayed until postoperative spinal bone fusion was achieved.
Lee et al.^30^	116	107 Male/9 Female (36.8±0.8^*^)	7.7±7.3 ^*^	NS	AAS; mean AADI=4.30±1.55 (mm±SD) AADI>4 mm (42 cases) Atlantoaxial ankylosis (9 cases)	2 patients underwent surgery (Fixation)	NS
Samartz-is et al.^31^	1	Male (61)	9	Hoffman and Babinski signs Hyperreflexia	Cord compression by a hypo plastic posterior arch of C1 with hyperextension	Excision of the C1 posterior arch for cord decompression	Significant postoperative improvement in numbness, spasticity and muscle strength. Progressive resolution of symptoms over a six-month period. Postoperative radiographs and MRI confirmed adequate decompression of the spinal cord. Recurrence of spinal cord compression at the foramen magnum following proximal migration of C2 after 10 years. Sudden death due to cardiorespiratory arrest.
Huang et al.^32^	1	Male (31)	6	Progressive weakness of the limbs	AAS; AADI=12 m Bone marrow oedema changes in the odontoid process Spinal cord compression and cervical myelopathy	Occipito-cervical fixation	Good clinical results at final follow-up
Zou et al.^33^	1	Male (24)	3	Cervical pain and limbs weakness Dyspnoea Decreased muscle strength throughout the limbs (Grade 2 of 5)	Rotatory atlantoaxial dislocation Narrowing of the spinal canal at the level of C1 Cervicomedullary compression	Halo dependent traction followed by occipito-cervical fixation	Postoperative improvement in muscle strength and sensory function. After a two-year follow-up, walking with a walker, with a favourable radiological outcome.
Shao et al.^34^	1	Male (44)	20	Limbs weakness Hyperreflexia Hoffman and Babinski signs	Posterior atlantoaxial dislocation Discontinuity of the dens Spinal cord injury	Occipito-cervical Fixation	Progressive improvement in neurological deficit postoperatively, with clear improvement following rehabilitation. Disappearance of neck pain and myelopathic symptoms at the 2-year follow-up.
Hadhri et al.^35^	1	Female (24)	5	Chronic cervical dystonia Fixed head rotation	Right rotatory atlantoaxial dislocation with 3,5mm anterior displacement of the atlas	Occipito-cervical Fixation	NS
Our case	1	Male (30)	Concomitant	9-month history of low back and neck pain Acute severe neck pain associated with paraesthesia in arms and legs Limited range of motion of the cervical spine	AAS; AADI=15mm Retraction of the central spinal canal with compression of the spinal cord at the C1-C2 level Bilateral ankylosis of the sacroiliac joints and a bamboo spine	Posterior decompression by suboccipital craniectomy and C1-C2 laminectomy. Occipitocervical fixation with placement of a bone graft taken from the iliac crest. Cervical collar for 3 months .	Improvement of symptoms postoperatively. Start of infliximab with improvement in AS activity.

NS: not specified; AS: Ankylosing Spondylarthritis; AAD: Atlantoaxial dislocation; AADI: Anterior Atlanto-Dental Interval; AAS: Anterior atlantoaxial subluxation.

* data are expressed as means + standard deviation.

The mean age was 34.43 ± 12.96 years, ranging from 18 to 61 years. The mean disease duration was 6.89 ± 8.66 [0–30] years. The AAD was anterior in 118 cases, rotatory in 5 cases, lateral in 1 case, and posterior in 1 case. An atlantoaxial ankylosis was found in 9 cases.

Clinical manifestations were neck pain (12 cases), neck stiffness (4 cases), fixed rotation of the head with limited range of motion (6 cases), muscle dystonia (2 cases), weakness and numbness of the limbs (7 cases), and dyspnoea in one case. Specific neurologic signs were found in 4 patients: Hoffman and Babinski signs in 3 cases, deep tendon reflexes hyperreflexia in 3 cases, nerve palsy in 1 case, and quadriparesis in 1 case. Clinical data were not detailed in one study.^30^ Luxation revealed AxSpA in 4 cases.^28^ MRI was performed in 8 cases, showing spinal cord compression in 4 cases, bone marrow oedema of the odontoid dens in 3 cases, periodontoid pannus in 2 cases, soft tissue oedema in 1 case, cervical myelopathy in 1 case, discontinuity in the dens in 1 case, and spinal cord injury in 1 case.

As for the treatment, surgery was indicated in 18 cases, consisting of occipito-cervical fixation in 9 cases, atlantoaxial fixation in 5 cases, and fixation with an unspecified level in 2 cases. In addition, one patient had a partial myectomy of the right sternocleidomastoid muscle and selective posterior ramisectomy of C1–C6 including C2 ganglionectomy on the left side, and another an excision of the C1 posterior arch for cord decompression. Traction was indicated in 3 patients, followed by occipito-cervical fixation in 2 cases and C1-C2 fixation in 1 case. Head and neck fixation with a plaster cast followed by a cervical collar was used in 1 case.

Symptom alleviation and favourable radiological findings were reported in 13 and 5 cases, respectively. However, one patient retained residual numbness of the upper limb and another an unchanged rotational fixation of the head.

One death was reported ten years after successful surgery. It was due to a recurrence of spinal cord compression at the foramen magnum following proximal C2 migration, which was responsible for cardiorespiratory arrest. Outcomes were not specified in 121 cases.

## DISCUSSION

The involvement of the atlantoaxial joint, especially the AAD, has been rarely reported in AxSpA patients.^34^ The atlantoaxial joint ensures most of the movements of the cervical spine. This joint consists of three synovial joints: a medial joint (atlanto-odontoid joint) and two lateral joints (facet joints). The stability of the atlanto-odontoid joint against anterior translation is provided by the transverse ligament.^36^Destruction or a loosening of this ligament leads to instability. Atlantoaxial instability, also known as AAD,^6^ can result from AAS, subaxial subluxation, rotatory subluxation,^37^ lateral subluxation,^38^ or vertical subluxation.^39^ The latter includes cranial settling (CS), vertical migration, atlantoaxial impaction and basilar invagination, corresponding to a projection of the tip of the odontoid process above the foramen magnum.^40^ Wang et al. had classified AAD into 4 types: instability, reducible dislocation, irreducible dislocation, and bony dislocations.^41^

AxSpA-related AAD can be explained by transverse ligament enthesitis^42^ and periodontoid pannus formation.^43^ Indeed, pannus formation can spread to the surrounding tissues, causing skeletal and ligament destruction, leading to dislocation.

Halla et al. also suggested the role of erosive synovitis of the facet joint in lateral mass collapse, which can induce excessive stress on the atlantoaxial joint, leading to AAD.^44^

ADD may precede,^23^ reveal,^45^ or occur during the follow-up of AxSpA patients. And although most patients with this condition remain asymptomatic,^46^ 50% of them complain of neck pain or restricted neck movement^47^ as it was the case for our patient.

Although our patient did not present any neurological abnormalities or deficits at the time of diagnosis, signs and symptoms of myelopathy, such as paraesthesia, numbness and weakness of the limbs, as well as bladder and bowel disturbances, have been described.^48^ These patients may also complain of crepitus or a sensation of the head “falling forward” during flexion.^49^ Cases of lower cranial nerve dysfunction, respiratory distress,^50^ quadriplegia, or death if left untreated^36^ have also been reported.

AAS is the most common C1-C2 dislocation observed in AxSpA patients^51^ as it was the case for our patient. It is defined as anterior anterior atlanto-dental interval (AADI) greater than 3 mm for adults and 5 mm for children.^52^ AADI higher than 8 mm denotes complete rupture of the transverse and alar ligaments.^53^ The AADI is usually measured on a dynamic cervical spine radiograph and corresponds to the horizontal distance between the posterior cortex of the anterior arch of the atlas and the anterior cortex of the dens in the median plane.^54,55^

Although dynamic radiographs may reduce the false-negative diagnosis rate, the diagnosis sensitivity of radio-graphs remains low.^56^ MRI has been shown to be more sensitive than radiographs. Indeed, Hung et al.^43^ found that MRI allows the diagnosis of AAS in 38% of patients (15 of 40) with normal radiographic AADI.

The latter authors also demonstrated that the combination of periodontal effusion, lateral facet arthropathy, abnormal intramedullary signal intensity, and an abnormal spinolaminar line had a sensitivity of 100% and a specificity of 90% for the diagnosis of AAS.^43^ Moreover, MRI can provide important biomechanical clues, 3D information and excellent soft tissue contrast.^57^

Rotatory dislocation, rarely described in AxSpA patients, may be responsible for reducible or fixed head tilt, neck pain, and occipital neuralgia. It is caused by unilateral involvement of the C1-C2 joint and abnormalities of the transverse ligament. Open-mouth radiographs may reveal lateral displacement of the dens and asymmetry of the C1 lateral masses. CT scan and MRI provide a more accurate assessment of this dislocation.^58^

Longer disease duration^35^ and peripheral arthritis^27,30,59^ were associated with an elevated risk of AAD. Furthermore, Lee et al. showed a significant association of AAS with elevated CRP (Odds Ratio (OR)=2.19, 95% Confidence Interval (_95%_CI) = [1.36–3.53]), peripheral arthritis (OR=2.05, _95%_CI = [1.36–3. 07]), use of a tumour necrosis factor inhibitors due to failure of NSAIDs or disease-modifying antirheumatic drugs (OR=2.28, 95%CI=[1.52–3.42]), and uveitis (OR=1.71, _95%_CI=[1.13–2.59]).^30^

If left untreated, AAD can lead to poor clinical outcomes, morbidity, and even death. Sunahara et al. studied 21 rheumatoid arthritis patients with myelopathy resulting from AAD who had refused surgery and had found a cumulative probability of survival of 0% during the first 7 years after the onset of myelopathy.^60^ Moreover, incompetence of the lateral masses of C1 could lead to vertical migration of the dens and CS, thus decreasing the distance between the odontoid process and the cranial cavity and leading to serious neurological complications.^61,62^

The treatment of AxSpA related AAD is not codified. Surgical treatment of patients with symptomatic AAD is widely indicated,^63^ especially in cases of intractable pain affecting daily activities,^28,64^ neurologic deficits,^28^ or vertical subluxations compromising the vertebral artery.^65^ Surgery aims to establish spinal stability and prevent neurological deterioration and spinal cord injury, thereby improving neurological function.^64^ Numerous studies have shown that early surgical intervention leads to more satisfactory outcomes.

Surgical treatment of anterior AAD is based on C1-C2 arthrodesis. There are several methods of atlantoaxial fusion. Wiring with the bone grafting technique was first described,^60^ then this method was revised.^66^ Bilateral C1 lateral mass and C2 pedicle screw fixation is the most used method.^67^ For our patient, we performed posterior decompression, and fusion with occipital plates, lateral mass screws, and a large craft harvested from the posterior iliac crest. We fixed this graft with a screw to the occiput. This approach diverges from the standard C1-C2 arthrodesis commonly used for anterior atlantoaxial dislocation.

Surgical treatment of posterior dislocation can include occipito-cervical arthrodesis^68,69^ or a combination of cervical screws and occipital plating.^70^ A C1 laminectomy may be required to relieve dorsal compression caused by persistent pannus, in which case an occipito-cervical fusion may be necessary.^71^ Posterior occipito-cervical arthrodesis may also be indicated in cases of rotatory subluxation.^25^ Odontoidectomy and ventral decompression can be proposed when AAD is complicated by CS.^72^ The therapeutic management of asymptomatic patients remained controversial. Some authors recommend a prophylactic arthrodesis in patients with a posterior atlanto-dental interval lower than 14 mm on lateral neck flexion radiographs and a space around the cord lesser than 13 mm on MRI.^73^

Conservative treatment may rarely be proposed in the absence of neurological deficit. It may include cervical halter traction in supine position with active range-of-motion exercises for 24 to 48 hours initially, followed by ambulatory orthotic immobilisation with active range-of-motion exercises,^74^ NSAIDs,^75^ or tumour necrosis factor inhibitors.^76^

This systematic review emphasises clinical characteristics, radiological features, risk factors, and therapeutic management of AAD in AxSpA patients. We performed a relevant MEDLINE search and hand-searched for relevant articles.

However, our study has some limitations. Publication bias was the major bias in our study. Indeed, we only included studies. We did not search for unpublished work. Moreover, the follow-up was not specified in most included articles.

## CONCLUSION

AAD has been found to be a non-rare complication in patients with AxSpA, regardless of cervical symptoms. Fortunately, most patients with AAD remain asymptomatic. However, a clear evaluation is important because this condition can lead to serious and life-threatening complications.

Therefore, patients with AxSpA should be regularly assessed radiologically for AAD, especially in cases of long course of AS, peripheral involvement, high disease activity, or failure of conventional therapy.

## Data Availability

The datasets used and/or analysed during the current study are available from the corresponding author upon reasonable request.
